# Symptom Diary–Based Analysis of Disease Course among Patients with Mild Coronavirus Disease, Germany, 2020

**DOI:** 10.3201/eid2705.204507

**Published:** 2021-05

**Authors:** Patricia Nicole Wiegele, Iyad Kabar, Laura Kerschke, Christopher Froemmel, Anna Hüsing-Kabar, Hartmut Schmidt, Elena Vorona, Richard Vollenberg, Phil-Robin Tepasse

**Affiliations:** University Hospital Muenster Department of Medicine B for Gastroenterology, Hepatology, Endocrinology, and Clinical Infectiology, Muenster, Germany (P.N. Wiegele, I. Kabar, C. Froemmel, A. Hüsing-Kabar, H. Schmidt, E. Vorona, R. Vollenberg, P.-R. Tepasse);; University of Muenster Institute of Biostatistics and Clinical Research, Muenster (L. Kerschke)

**Keywords:** COVID-19, pneumonia, acute respiratory distress syndrome, ARDS, symptom diary, symptoms, coronavirus disease, SARS-CoV-2, severe acute respiratory syndrome coronavirus 2, viruses, respiratory infections, zoonoses, Germany

## Abstract

Limited information is available on the clinical course of outpatients with mild coronavirus disease (COVID-19). This information is critically important to inform public health prevention strategies and to provide anticipatory guidance to patients, primary care providers, and employers. We retrospectively assessed the daily prevalence of symptoms in 313 COVID-19 outpatients for the first 20 days of illness. Generalized estimating equations were used to assess the probability of symptom occurrence over time. Fatigue (91%), cough (85%), and headache (78%) were the most common symptoms and occurred a median of 1 day from symptom onset. Neurologic symptoms, such as loss of taste (66%) and anosmia (62%), and dyspnea (51%) occurred considerably later (median 3–4 days after symptom onset). Symptoms of COVID-19 are similar to those of other respiratory pathogens, so symptomatic patients should be tested more frequently for severe acute respiratory syndrome coronavirus 2 during influenza season to prevent further spread of COVID-19.

Coronavirus disease (COVID-19) is a highly contagious disease caused by severe acute respiratory syndrome coronavirus 2 (SARS-CoV-2) (*1*). SARS-CoV-2 was first identified in December 2019 in Wuhan, China, and quickly spread across the world (*2*). At the beginning of the COVID-19 pandemic, studies mainly focused on the epidemiologic and clinical characteristics of hospitalized and critically ill patients (*3*–*6*). Fever, cough, and dyspnea were identified as the most common symptoms in critically ill patients (*7*,*8*). Chemosensory symptoms, including loss of taste and smell, were highly prevalent in mildly ill patients and thus more common in COVID-19 than in other respiratory viral diseases (*9*,*10*). Male sex, older age, obesity, and underlying conditions such as diabetes and cardiovascular disease are risk factors for severe or fatal disease (*11*–*13*). As the pandemic has spread worldwide, the numbers of COVID-19 outpatients with mild clinical manifestations have increased steadily, and such patients currently represent ≈80% of all confirmed cases (*14*). To prevent further spread of SARS-CoV-2, detecting such cases early is essential because both asymptomatic and oligosymptomatic patients can transmit the virus (*15*). To help in early identification of mild SARS-CoV-2 infections, we investigated symptom prevalence and severity on a daily basis in COVID-19 patients with a mild disease course.

## Methods

### Study Population

In March 2020, the University Hospital of Muenster (Muenster, Germany) started outreach to the public to identify persons who recovered from SARS-CoV-2 infection through press briefings and social media. The outreach did not imply any conditions for participation. A total of 2,136 persons who had recovered from SARS-CoV-2 infection reported back (by email, telephone, and mail), stated that the infection was confirmed by PCR testing of nasopharyngeal swab specimens, and reported their willingness to participate in further studies. They were asked for the availability of an individual symptom diary by email. Among the 2,136 case-patients, 736 stated that they kept a detailed symptom diary during the disease course. These 736 participants received a detailed online questionnaire inquiring retrospectively on a daily basis about COVID-19 symptom prevalence, severity, duration, and timing. By filling out the online questionnaire, participants transferred their own symptom diary to the online questionnaire and provided structured data for further analyses. The time interval from positive pharyngeal swab specimen test result to filling out the online questionnaire was 8–12 weeks. The study was approved by the Ethics Committee of Muenster University. All patients provided written informed consent.

### Epidemiologic and Clinical Data

In the online questionnaire, 736 participants were asked for the date when their first COVID-19 symptoms occurred. This date was defined as baseline (day 1). Within 20 days of symptom onset, participants had to indicate on a daily basis the presence or absence of various predefined symptoms of COVID-19 according to current literature (*16*,*17*). For every single day and every single symptom (Table 1), patients had to choose from a dropdown menu between absence versus presence. In case of presence of abdominal pain, nausea, loss of taste, vision disorders, hearing loss, loss of smell, cough, rhinitis, sore throat, myalgia, headache, and fatigue, participants further had to rate the intensity on a numeric rating scale (NRS) from 0 to 10 by using a dropdown menu. NRS data were classified into 5 different symptom severity grades: grade 0 (NRS 0), grade 1 (NRS 1–2), grade 2 (NRS 3–5), grade 3 (NRS 6–8), and grade 4 (NRS 9–10). Severity of dyspnea was measured using the 5-point modified Medical Research Council dyspnea scale from 0 to 4 (*18*). The indication of fever was based on a subjective assessment. Skin lesions (alterations of any kind in the area of the skin), mucosal lesions (alterations of any kind in the area of the mucous membrane), and vision disorders were not further specified in the questionnaire. Symptom prevalence indicates the number of participants who experienced a particular symptom at least once during the entire illness. If a symptom persisted for longer than 20 days, participants had to indicate the date when they experienced the symptom for the last time. When calculating the median symptom onset, we included in the analysis only persons who experienced the symptom >1 day during the illness. In addition, participant demographic characteristics, including age, sex, and body mass index (BMI) were collected. Among the 736 case-patients who received the questionnaire, 332 completed the questionnaire in its entirety. Nineteen persons reported hospitalization during the disease course and were excluded. Data from 313 persons were included in further analyses.

**Table 1:** COVID-19 symptom characteristics in 313 patients participating in a symptom diary–based analysis of COVID-19 disease course, Germany, 2020*

Symptom	Prevalence, no. (%)†	Median day of onset (minimum–maximum)‡
Fatigue	285 (91.1)	1.0 (1–18)
Cough	266 (85.0)	1.0 (1–15)
Headache	244 (78.0)	1.0 (1–13)
Myalgia	229 (73.2)	1.0 (1–18)
Rhinitis	220 (70.3)	1.0 (1–20)
Loss of taste	208 (66.5)	4.0 (1–19)
Sore throat	204 (65.2)	1.0 (1–10)
Loss of smell	195 (62.3)	3.0 (1–19)
Fever	191 (61.0)	2.0 (1–20)
Dysgeusia	162 (51.8)	4.0 (1–2)
Dyspnea	160 (51.1)	3.0 (1–15)
Loss of appetite	140 (44.7)	3.0 (1–13)
Dizziness	126 (40.3)	2.0 (1–20)
Diarrhea	102 (32.6)	4.0 (1–20)
Nausea	100 (31.9)	3.0 (1–20)
Abdominal pain	88 (28.1)	2.5 (1–20)
Hearing loss	62 (19.8)	3.0 (1–16)
Vision disorders	58 (18.5)	3.0 (1–20)
Mucosal lesions	43 (13.7)	2.0 (1–12)
Skin lesions	31 (9.9)	6.0 (1–13)
Vomiting	10 (3.2)	5.0 (1–11)

*COVID-19, coronavirus disease. †Symptom occurrence within the observation period was based on the overall cohort. Results shown as absolute and relative frequencies. †Day of symptom onset. Calculations of median days to symptom onset were restricted to patients who reported experiencing that symptom.

### Statistical Analysis

We performed statistical analyses by using SPSS Statistics 26 for Macintosh (IBM, https://www.ibm.com) and R version 3.6.0 (R Project for Statistical Computing, https://www.r-project.org). Inferential statistics were intended to be exploratory (i.e., hypothesis generating). We interpreted p values as a metric weight of evidence against the respective null hypothesis of no effect, and no adjustment for multiple testing was made; p values <0.05 were considered statistically significant. We analyzed patient and symptom characteristics using standard descriptive statistics. We presented normally distributed continuous variables as means + SDs, minima, and maxima, categorical variables as counts and relative frequencies, and non–normally distributed continuous variables as medians, minima, and maxima. For each COVID-19 symptom, generalized estimating equations were used to assess the effect of time since symptom onset, age, sex, and BMI on the odds of being affected by the symptom. To account for the nonlinear relationship between time since symptom onset and symptom presence, the models also included a quadratic effect of time. Dependencies between longitudinal measurements in the same patient were modeled by a first-order autoregressive correlation structure. Results were reported as odds ratios (ORs), corresponding 95% CIs, and p values. An ordinal regression analysis based on proportional odds cumulative logit models was performed to evaluate the association between the maximum intensity of the symptoms loss of taste and loss of smell (because these manifestations are more common in COVID-19 than in other respiratory viral diseases) that was observed within the 20-day period of symptom onset and the independent parameters age, sex, and BMI. The symptom intensity was modeled on the basis of all patients who were affected by the respective symptom. Results are reported as ORs, 95% CIs, and p values.

## Results

### Characteristics of Study Participants

A total of 313 participants completely filled out the online questionnaire and were included in the analyses. We summarized the characteristics of the study population (Table 2).

**Table 2:** Characteristics of 313 COVID-19 patients participating in a symptom diary–based analysis of COVID-19 disease course, Germany, 2020*

Characteristic	No. (%)
Age, y, mean + SD (range)	45.5 ± 13.1 (17–92)
Sex	
M	142 (45.4)
F	171 (54.6)
BMI, mean + SD (range)	24.7 ± 4.2 (17.7–46.3)
Underlying condition	
Diabetes mellitus	5 (1.6)
Cardiovascular disease	36 (11.5)
Liver disease	3 (1.0)
Thyroid disease	18 (5.8)
Pulmonary disease	20 (6.4)
Hospitalization	0
Invasive ventilation	0
Oxygen supply	0
Intensive-care unit	0

*Values are no. (%) except as indicated. BMI, body mass index; COVID-19, coronavirus disease.

### First Appearance of COVID-19 Symptoms

Fatigue (91.1%), cough (85.0%), and headache (78.0%) were the most common symptoms and occurred within a median of 1 day after symptom onset. Further common general symptoms were myalgia (73.2%), rhinitis (70.3%), and sore throat (65.2%), occurring within a median of 1 day. Fever was reported by 61% of study participants within a median of 2 days after symptom onset. Symptoms of the lower respiratory tract (dyspnea) were reported by 51.1% of all participants and occurred within a median of 3 days after symptom onset, notably later than most other symptoms. The first appearance of neurologic symptoms including loss of taste (66.5%), dysgeusia (51.8%), and loss of smell (62.3%) was reported within a median of 3–4 days after symptom onset, also notably later than most other symptoms. Gastrointestinal symptoms including nausea (31.9%), vomiting (3.2%), and diarrhea (32.6%) also occurred notably later, within a median of 3–5 days.

### Prevalence of COVID-19 Symptoms over Time

**Figure 1:**
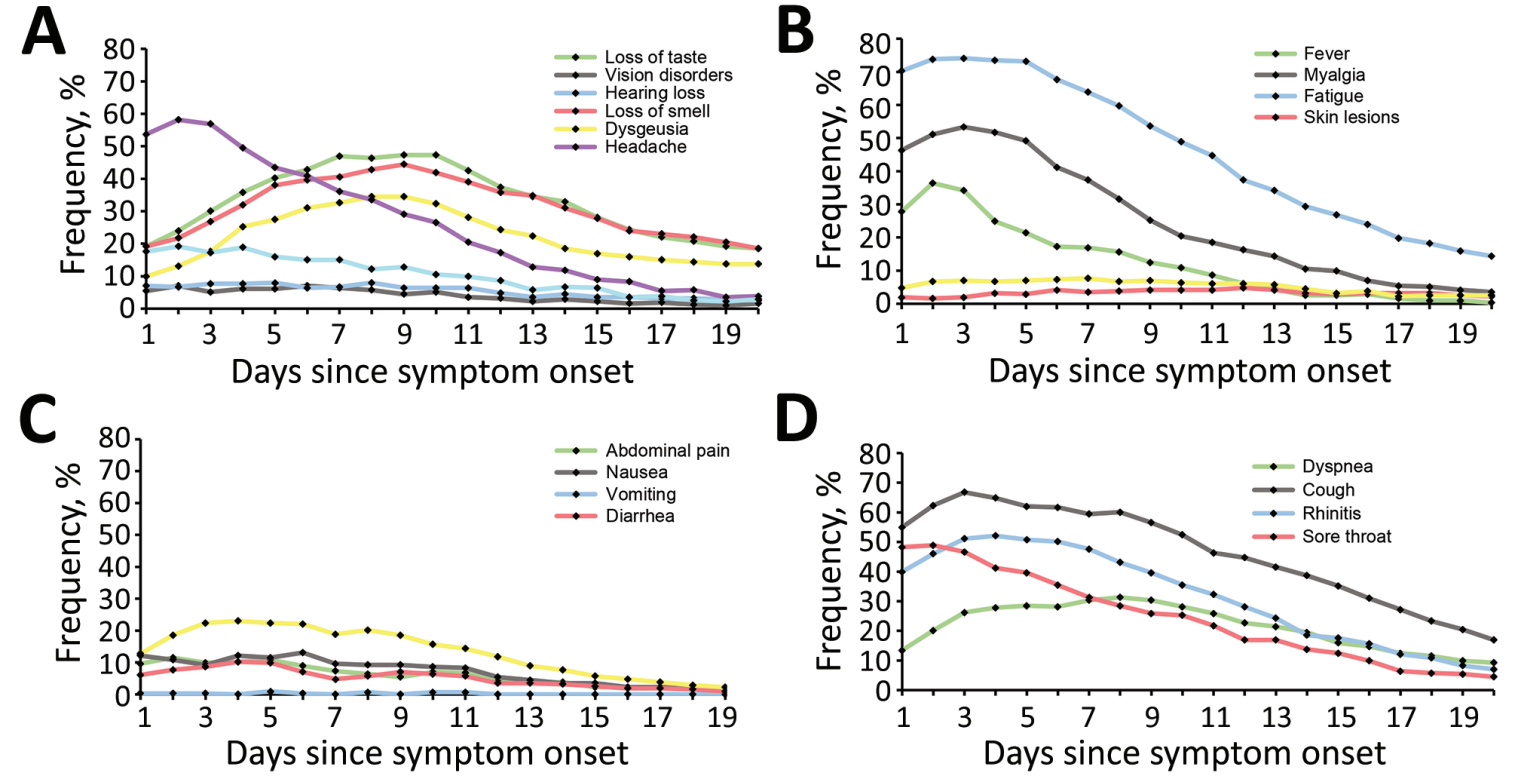
Prevalence of coronavirus disease symptoms over time among 313 patients participating in a symptom diary–based analysis of disease course, Germany, 2020. Line graphs show the occurrence of neurologic symptoms (A), general symptoms (B), gastrointestinal symptoms (C), and respiratory symptoms (D) within 20 days of symptom onset.

We compiled the daily prevalence of neurologic, general, gastrointestinal, lower respiratory, upper respiratory, and dermatologic symptoms within 20 days of symptom onset (Figure 1). Neurologic symptoms (Figure 1, panel A), such as dysgeusia, loss of taste, and loss of smell, had almost identical and quadratically shaped prevalence time courses. Within the first week of symptom onset, the number of participants affected by these symptoms increased rapidly. Dysgeusia reached the maximum on days 8 and 9 (34.5%), loss of taste on days 9 and 10 (47.3%), and loss of smell on day 9 (44.4%). General symptoms such as fever, myalgia, and fatigue (Figure 1, panel B) were frequently present from the beginning of COVID-19 symptom onset. The prevalence of these symptoms increased only slightly within the first days. Fatigue peaked on day 3 (74.1%), fever on day 2 (36.4%), and myalgia on day 3 (53.4%). Gastrointestinal symptoms showed a flat curve in symptom occurrence over time (Figure 1, panel C). The prevalence of upper respiratory symptoms, such as rhinitis, cough, stand sore throat, peaked during days 1–4, whereas the prevalence of a lower respiratory symptom (dyspnea) reached its maximum on day 8 (33.3%) (Figure 1, panel D). 

Results from the generalized estimating equation analysis assessed the effect of time, age, sex, and BMI on symptom presence (Appendix Table). Age was positively associated with the odds of hearing loss, general symptoms (fatigue, fever, and myalgia), gastrointestinal symptoms (nausea, abdominal pain, and loss of appetite), and respiratory symptoms (dyspnea and cough) (ORs 1.02–1.05; p<0.05). Women were more likely than men to be affected by neurologic symptoms (dysgeusia, loss of taste, loss of smell, headache, dizziness, and vision disorders), fatigue, myalgia, skin lesions, diarrhea, loss of appetite, rhinitis, and dyspnea (ORs 1.41–2.95; p<0.05). The odds of all symptoms except for fever, mucosal lesions, skin lesions, sore throat, diarrhea, and loss of appetite increased with increasing BMI (ORs 1.05–1.11; p<0.05). 

**Figure 2:**
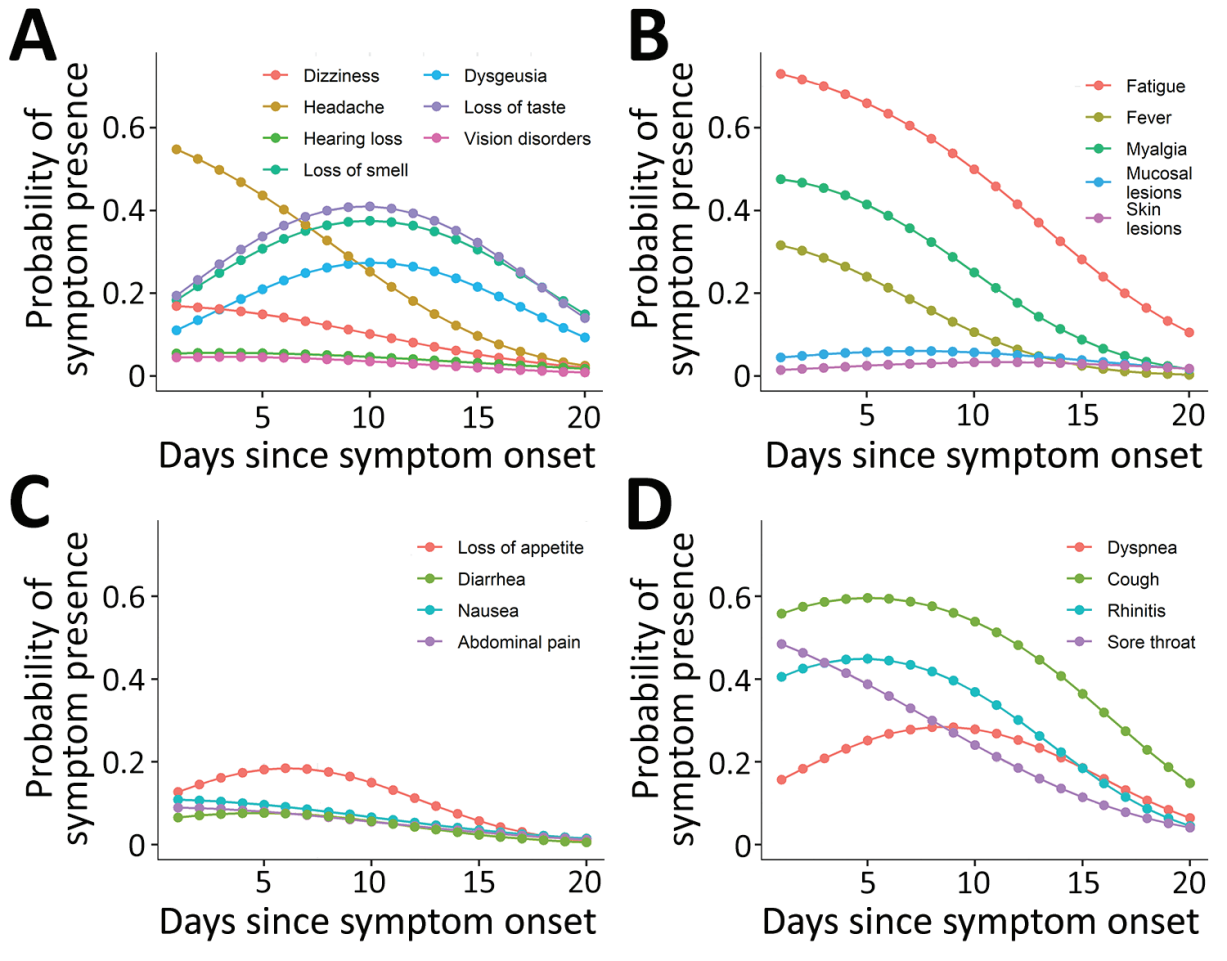
Probability of symptom presence over time among 313 coronavirus disease patients participating in a symptom diary–based analysis of disease course, Germany, 2020. Probabilities derived from the generalized estimating equation analysis for neurologic (A), general and dermatologic (B), gastrointestinal (C), and respiratory symptoms (D).

In line with the descriptive analysis of daily symptom prevalence we have outlined, the odds of the presence of most symptoms showed a quadratic trend over time since symptom onset (ORs of the quadratic effect of day 0.98–0.996; p<0.05). The quadratic trends were characterized by an initial increase and a subsequent decrease in the odds of dysgeusia, loss of taste, loss of smell, hearing loss, mucosal lesions, loss of appetite, diarrhea, nausea, cough, rhinitis, and dyspnea. For all other symptoms, we observed an almost linear monotonic decrease in the odds of symptom presence over time (Figure 2).

### Maximum Intensity of COVID-19 Symptoms

**Figure 3:**
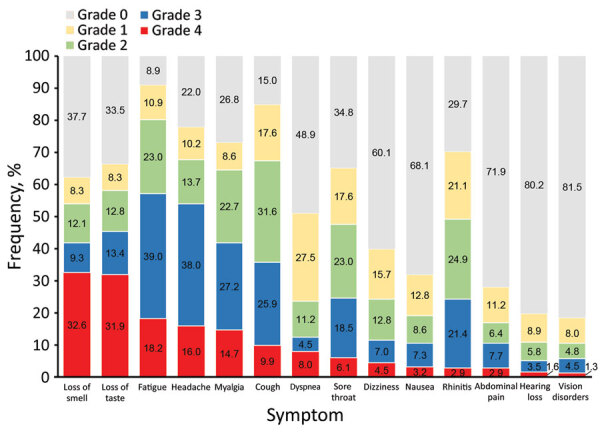
Maximum severity of coronavirus disease symptoms within 20 days of symptom onset among 313 patients participating in a symptom diary–based analysis of disease course, Germany, 2020. Bar plots show the frequencies of all participants experiencing symptoms of intensity grade 0 (none), grade 1 (mild), grade 2 (moderate), grade 3 (severe), or grade 4 (maximum imaginable) within 20 days of symptom onset. For each patient, the highest reported intensity in the 20-day period was chosen.

For the overall cohort, Figure 3 shows the distribution of maximal symptom intensity by severity grades 0–4 occurring within 20 days of COVID-19 symptom onset. Fatigue (57.2%), headache (54.0%), loss of taste (45.3%), loss of smell (41.9%), and myalgia (41.9%) were most frequently reported with severity grades 3 and 4.

For the overall cohort, Figure 3 shows the distribution of maximal symptom intensity by severity grades 0–4 occurring within 20 days of COVID-19 symptom onset. Fatigue (57.2%), headache (54.0%), loss of taste (45.3%), loss of smell (41.9%), and myalgia (41.9%) were most frequently reported with severity grades 3 and 4.

We determined that multiple factors were associated with severe symptom intensity within 20 days after symptom onset among all study participants (Table 3). Women were found to be at increased risk for having a severe course of loss of taste (OR 2.796 [95% CI 1.35–5.88]; p = 0.006) and loss of smell (OR 2.694 [95% CI 1.53–4.78]; p = 0.001). Age was negatively associated with the maximum symptom severity of smell loss (OR 0.968 [95% CI 0.95–0.99]; p = 0.004).

**Table 3:** Factors associated with severe COVID-19 symptom intensity within 20 days of symptom onset among 313 COVID-19 patients participating in a symptom diary–based analysis of COVID-19 disease course, Germany, 2020*

Symptom	Age			Female			BMI	
	OR (95% CI)	p value		OR (95% CI)	p value		OR (95% CI)	p value
Loss of taste	0.977 (0.95–1.01)	0.117		2.796 (1.35–5.88)	0.006		0.965 (0.89–1.05)	0.385
Loss of smell	0.968 (0.95–0.99)	0.004		2.694 (1.53–4.78)	0.001		1.000 (0.93–1.08)	0.989

*BMI, body mass index; COVID-19, coronavirus disease; OR, odds ratio..

### Time Course of COVID-19 Symptom Intensity

**Figure 4:**
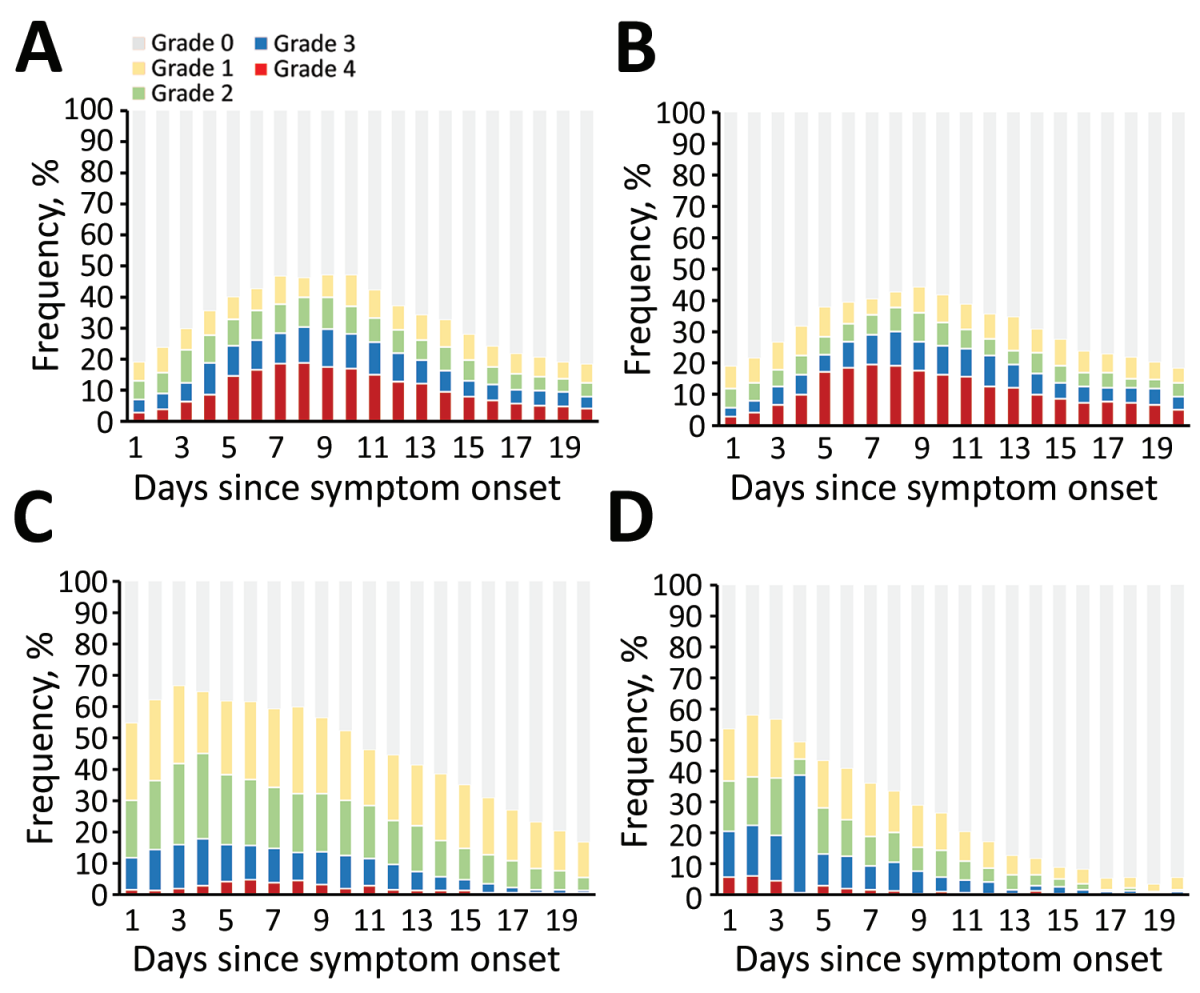
Time course of coronavirus disease symptom severity among 313 patients participating in a symptom diary–based analysis of disease course, Germany, 2020. Bar charts show the distributions of symptom severities: grade 0 (none), grade 1 (mild), grade 2 (moderate), grade 3 (severe), or grade 4 (maximum imaginable). Severity of loss of taste (A), loss of smell (B), cough (C), and headache (D) were evaluated over 20 days from symptom onset.

We assessed the distributions of symptom intensity grades reported within 20 days of COVID-19 symptom onset (Figure 4). Loss of taste and loss of smell were characterized by relatively high rates of grade 3 and 4 symptom severities. These symptoms showed a steady increase within the first week of symptom onset and reached maximums on day 8 (30.4% for loss of taste, 30.0% for loss of smell). In comparison, cough and headache showed less severity, with a steady decrease in intensity during the first week, except for day 4, when a notable increase in grade 3 headaches occurred compared with day 3 (14.7% vs. 38.0%).

## Discussion

Our study examined the daily prevalence and severity of COVID-19 symptoms occurring within 20 days of symptom onset in mildly ill outpatients and revealed new insights in symptom development during the disease course. Fatigue, cough, chemosensory disorder, and dyspnea were highly prevalent in mild COVID-19. We were able to show that lower respiratory and chemosensory symptoms, which are considered more characteristic of COVID-19, appear significantly later than general and nonspecific symptoms such as fatigue and upper respiratory symptoms. Results of our study highlight the positive associations of BMI, age, and especially female sex with the frequency of characteristic disease symptoms.

In line with previous studies, we showed that chemosensory symptoms such as smell and taste disorders were highly prevalent among mildly ill COVID-19 patients, usually occurring 3–4 days after symptom onset (*19*–*23*). Both the number of patients affected by these symptoms and the proportion of patients experiencing these symptoms with severe intensity increased steadily during the first week, suggesting progressive central nervous system involvement. In our study, the appearance of neurologic symptoms (except for headache) was delayed compared with general and respiratory manifestations.

Gastrointestinal symptoms (nausea, vomiting, and diarrhea) also occurred notably later, within a median of 3–5 days, compared with general and upper airway symptoms, which is consistent with previous studies (*24*,*25*). Nobel et al. (*26*) showed an increased probability of SARS-CoV-2 infection in patients who had gastrointestinal symptoms in addition to further symptoms characteristic of COVID-19, compared with patients without gastrointestinal symptoms; these findings and our data suggest that physicians must be aware of SARS-CoV-2 infection in case of coexisting general and gastrointestinal symptoms. Nevertheless, because gastrointestinal symptom onset appears later in disease, the risk for misdiagnosis at the very beginning of disease might be increased.

In our cohort, general and upper airway symptoms were prominent early indications of COVID-19, occurring within a median of 1 day after symptom onset. At symptom onset, fatigue (70.3%) and cough (55.0%) were the most frequently observed symptoms. In contrast, chemosensory and lower pulmonary symptoms (e.g., dyspnea), which are considered characteristic of COVID-19 (*27*,*28*), occurred within a median of 3–4 days after symptom onset and peaked during the second week. Our results show that multiple characteristic symptoms, including loss of smell (19.2% vs. 44.4%), dyspnea (13.4% vs. 31.3%), loss of taste (19.2% vs. 47.3%), and dysgeusia (9.9% vs. 34.5%), rarely occurred on the first day of COVID-19 symptom onset compared with the day of maximal frequency. In agreement with other studies, we found a delay of up to 1 week between the emergence of upper respiratory symptoms and pulmonary manifestations, including dyspnea (*16*,*17*). This finding might indicate that viral movement from the upper airway to deeper levels occurs within a week. The late development of more specific symptoms, such as dyspnea, loss of taste, dysgeusia, and loss of smell, might lead to delays in diagnosis, especially during the season of seasonal colds and influenza, because of increased occurrence of seasonal pathogens producing nonspecific symptoms (*29*–*32*). Our data highlight a critical period of up to 4 days after symptom onset with the potential for delayed diagnosis and further disease spread; infected persons can be highly contagious within 2 days before symptom onset and up to 10 days thereafter (*33*–*35*). Our data are in line with data from Yousaf et al. (*21*), who prospectively analyzed symptom profiles of 47 nonhospitalized household contacts with SARS-CoV-2 infection. However, there are differences in methodology and results between that report and this one.

According to our data, female sex was strongly associated with the occurrence of neurologic symptoms. Furthermore, women were more frequently affected by fatigue, myalgia, skin lesions, diarrhea, loss of appetite, rhinitis, and dyspnea. In line with our findings, Lechien et al. (*36*) reported that loss of smell, headache, and fatigue were significantly more prevalent in women. We extend these findings by showing that women suffer loss of taste and loss of smell with higher intensity during the first 20 days after symptom onset compared with men (Table 3).

In our study, higher BMI was significantly associated with the occurrence of general symptoms, respiratory symptoms, and neurologic symptoms. In addition, we were able to show that in mildly affected patients the likelihood of having onset of hearing loss, gastrointestinal symptoms, general symptoms, and respiratory symptoms increases with age. In contrast to Lee et al. (*37*), we could not prove that young participants were more often affected by loss of smell, but we found that they suffer this symptom with a higher intensity during the first 20 days after symptom onset. These findings suggest that older patients infected with SARS-CoV-2 are more likely to have general COVID-19 symptoms than specific symptoms such as loss of smell, potentially increasing the risk for misdiagnosis.

Our study’s first limitation is that using social media and press briefings could have biased our results toward young persons who use social media and would be exposed to the press briefings. Using online symptom diaries might have limited participation by less technologically literate persons, persons without internet at home, and persons who did not record symptom diaries or have time to complete the online form. Second, recall bias might have affected results. Participants kept an individual symptom diary during disease and retrospectively transferred these data into the online questionnaire 8–12 weeks after onset of the first symptom. Patients might have added information that was not in their original diaries. In addition, they had to choose the absence or presence of predefined symptoms, and the predefinition might have influenced symptoms reported by participants. Third, the data evaluated in this study were based on subjective patient statements. Unlike some other studies, no validated chemosensory tests were performed (*38**,**39*). Fourth, only ≈50% of persons who were eligible participated, potentially affecting the representativeness of the findings.

We describe the probability of the occurrence of different symptoms in mild COVID-19 on a daily basis by analysis of information directly obtained from the patient in a large cohort with mild disease symptoms. Despite the retrospective design, data were well preserved by patient’s diaries, which might limit the potential recall bias we have described. To identify infected persons early in the disease course, exact knowledge of symptom prevalence in this period is very important, and our study provides useful data that could substantially improve early diagnosis of COVID-19.

In conclusion, our study found that general and upper airway symptoms appear soon after COVID-19 symptom onset in mild cases but lower respiratory tract and neurologic symptoms, both considered characteristic of COVID-19, occur significantly later. Older men might experience less frequent and less severe neurologic symptoms. Particularly in the season for seasonal colds and influenza, extreme caution is required in early identification of patients infected with SARS-CoV-2 because other seasonal viral diseases can initially produce very similar symptoms and because symptoms most characteristic of COVID-19 rarely occur on the first day of disease.

Appendix Time course of coronavirus disease symptoms assessed using generalized estimating equation analysis of disease course, Germany, 2020.
