# Supplementary material for: Symptom Diary–Based Analysis of Disease Course among Patients with Mild Coronavirus Disease, Germany, 2020
Source: Emerg Infect Dis. 2021 May;27(5):1353–61. doi: 10.3201/eid2705.204507 (PMC8084503; doi:10.3201/eid2705.204507)
Supplement: Appendix — Time course of coronavirus disease symptoms assessed using generalized estimating equation analysis of disease course, Germany, 2020. [file 20-4507-Techapp-s1.pdf]

Article DOI: https://doi.org/10.3201/eid2705.204507

Symptom Diary–Based Analysis of Disease Course among Patients with Mild Coronavirus Disease, Germany, 2020

Appendix

Appendix Table. Time course of COVID-19 symptoms assessed using generalized estimating equation analysis

	Day		Day^2†		Age‡		Female§		BMI¶	
Symptom*	OR (95% CI)	p value	OR (95% CI)	p value	OR (95% CI)	p value	OR (95% CI)	p value	OR (95% CI)	p value
Fatigue	0.950 (0.90–1.01)	0.092	0.995 (0.99–1.00)	<0.001	1.026 (1.01–1.04)	<0.001	2.306 (1.67–3.19)	<0.001	1.067 (1.03–1.11)	0.001
Cough	1.101 (1.04–1.16)	0.001	0.990 (0.99–0.99)	<0.001	1.019 (1.01–1.03)	0.005	1.238 (0.90–1.70)	0.190	1.062 (1.02–1.11)	0.003
Headache	0.928 (0.87–0.99)	0.024	0.994 (0.99–1.00)	0.001	1.003 (0.99–1.02)	0.608	1.408 (1.01–1.96)	0.044	1.065 (1.03–1.11)	0.001
Myalgia	0.997 (0.93–1.07)	0.927	0.990 (0.99–0.99)	<0.001	1.021 (1.01–1.04)	0.002	1.418 (1.01–1.98)	0.041	1.053 (1.01–1.10)	0.012
Rhinitis	1.126 (1.06–1.19)	<0.001	0.988 (0.99–0.99)	<0.001	0.988 (0.98–1.00)	0.047	1.424 (1.01–2.01)	0.046	1.059 (1.01–1.11)	0.014
Loss of taste	1.309 (1.24–1.39)	<0.001	0.986 (0.98–0.99)	<0.001	1.008 (1.00–1.02)	0.210	2.305 (1.61–3.30)	<0.001	1.046 (1.01–1.09)	0.022
Sore throat	0.928 (0.88–0.98)	0.010	0.996 (0.99–1.00)	0.007	0.997 (0.98–1.01)	0.647	0.980 (0.68–1.42)	0.916	1.048 (1.00–1.10)	0.058
Loss of smell	1.276 (1.20–1.35)	<0.001	0.988 (0.99–0.99)	<0.001	1.000 (0.99–1.01)	0.958	2.453 (1.69–3.57)	<0.001	1.059 (1.01–1.11)	0.011
Fever	0.972 (0.88–1.07)	0.573	0.989 (0.98–1.00)	0.001	1.019 (1.01–1.03)	0.004	0.807 (0.57–1.14)	0.221	1.029 (0.99–1.07)	0.131
Dysgeusia	1.311 (1.23–1.40)	<0.001	0.987 (0.98–0.99)	<0.001	1.006 (0.99–1.02)	0.387	2.516 (1.67–3.78)	<0.001	1.051 (1.01–1.10)	0.031
Dyspnea	1.257 (1.17–1.36)	<0.001	0.987 (0.98–0.99)	<0.001	1.019 (1.00–1.03)	0.014	2.218 (1.46–3.38)	<0.001	1.064 (1.01–1.12)	0.012
Loss of appetite	1.227 (1.12–1.34)	<0.001	0.983 (0.98–0.99)	<0.001	1.027 (1.01–1.04)	0.001	2.503 (1.62–3.88)	<0.001	1.044 (1.00–1.09)	0.051
Dizziness	0.996 (0.92–1.08)	0.927	0.994 (0.99–1.00)	0.013	1.006 (0.99–1.02)	0.510	1.680 (1.02–2.76)	0.040	1.076 (1.03–1.13)	0.002
Diarrhea	1.117 (0.98–1.27)	0.098	0.988 (0.98–1.00)	0.001	1.008 (0.99–1.03)	0.411	1.863 (1.11–3.13)	0.019	1.051 (0.98–1.12)	0.152
Nausea	0.996 (0.90–1.10)	0.940	0.995 (0.99–1.00)	0.063	1.027 (1.01–1.05)	0.003	1.763 (0.99–3.15)	0.055	1.074 (1.03–1.12)	0.002
Abdominal pain	1.000 (0.91–1.10)	0.993	0.995 (0.99–1.00)	0.059	1.022 (1.00–1.04)	0.024	1.846 (0.97–3.50)	0.060	1.063 (1.02–1.11)	0.004
Hearing loss	1.026 (0.94–1.12)	0.551	0.996 (0.99–1.00)	0.053	1.047 (1.03–1.07)	<0.001	1.536 (0.71–3.34)	0.279	1.110 (1.04–1.19)	0.002
Vision disorders	1.042 (0.93–1.16)	0.461	0.994 (0.99–1.00)	0.052	1.027 (1.00–1.06)	0.066	2.946 (1.16–7.49)	0.023	1.070 (1.01–1.13)	0.016
Mucosal lesions	1.125 (1.01–1.26)	0.040	0.992 (0.99–1.00)	0.003	0.983 (0.96–1.01)	0.250	2.335 (0.97–5.63)	0.059	1.050 (0.98–1.12)	0.152
Skin lesions	1.199 (1.03–1.40)	0.022	0.992 (0.99–1.00)	0.008	1.025 (1.00–1.06)	0.095	2.725 (1.11–6.70)	0.029	0.945 (0.85–1.05)	0.282

*The symptom “vomiting” was not included in the analyses because the frequencies of patients affected by these symptoms were too low. Vision disorders, skin lesions and mucosal lesions were not further specified.
†Day^2 = quadratic effect of day.
‡Age (x+1 vs. × years).
§Sex (female vs. male).
¶BMI (x+1 vs. × kg/m^2).
